# From Coherence to Multivariate Causal Estimators of EEG Connectivity

**DOI:** 10.3389/fphys.2022.868294

**Published:** 2022-04-26

**Authors:** Maciej Kaminski, Katarzyna J. Blinowska

**Affiliations:** ^1^ Department of Biomedical Physics, Faculty of Physics, University of Warsaw, Warsaw, Poland; ^2^ Nalecz Institute of Biocybernetics and Biomedical Engineering, Warsaw, Poland

**Keywords:** connectivity measures, effective connectivity, common drive effect, multivariate analysis, graph analysis, assortative mixing

## Abstract

The paper concerns the development of methods of EEG functional connectivity estimation including short overview of the currently applied measures describing their advantages and flaws. Linear and non-linear, bivariate and multivariate methods are confronted. The performance of different connectivity measures in respect of robustness to noise, common drive effect and volume conduction is considered providing a guidance towards future developments in the field, which involve evaluation not only functional, but also effective (causal) connectivity. The time-varying connectivity measure making possible estimation of dynamical information processing in brain is presented. The methods of post-processing of connectivity results are considered involving application of advanced graph analysis taking into account community structure of networks and providing hierarchy of networks rather than the single, binary networks currently used.

## Introduction

In the last years in the domain of brain research the problem of the determination of connectivity patterns has been a subject of intense studies. The assessment of coupling between brain structures is crucial for understanding of information processing in brain. The evidence about the activity of neural populations supplied by scalp EEG is limited and corrupted by noise and volume conduction, however an application of proper methods of analysis allows to extract from it a valuable information. The large repertoire of connectivity measures encompasses linear and non-linear, bivariate, and multivariate, directed and undirected methods.

We can distinguish anatomical, functional, and effective connectivity. Anatomical connectivity informs about a network of physical or structural connections linking sets of neurons. Herein we will not consider structural connectivity. The functional connectivity represents statistical interdependence between brain structures, it does not provide an information concerning the inference about the directed coupling between two brain regions. The effective connectivity determines causal dependence, it refers explicitly to the influence that one neural system exerts over another (Friston, 2011). When choosing a method of connectivity analysis we should take into account, if it represents the optimal approach in respect of solving of a given problem, if it is robust in respect of noise, common drive effect and volume conduction, and if it is not prone to arbitrary choices.

In the paper, first we shall consider bivariate, then multivariate measures of connectivity, with an impact on less popular multivariate methods based on Granger causality principle. Then we shall refer to time-varying connectivity. Next we shall consider performance of different connectivity measures in respect of noise, volume conduction and common source effect. The post-processing of results by means of Graph Analysis will be discussed. Finally we shall give some recommendations concerning application of different connectivity measures.

## Bivariate Measures of Connectivity

The most commonly used measures of connectivity are correlation and coherence. In case of EEG, which is characterized by distinct rhythms, it is coherence, which is commonly used. Ordinary (bivariate) coherence may be treated as a normalized cross-spectrum. It is a complex quantity. Its modulus measures the spectral power in a given frequency common to both signals. The phase of coherence is directly related to the time delay between signals. However the phase is ambiguous, since it is circular modulo 2π.

Phases of two signals may be synchronized, even if their amplitudes remain uncorrelated. This phenomenon is called phase synchronization. Several indexes of phase synchronization were introduced. Mean phase coherence, also called phase locking value (PLV) is computed as the length of the vector-average of a set of unit-length phase difference estimates. Still another measure, the phase locking Index (PLI) is a metric that evaluates the distribution of phase differences across observations. Phase Slope Index (PSI) is computed from the complex-valued coherence, and quantifies the consistency of the direction of the change in the phase difference across frequencies. The sign of the PSI informs which signal is temporally leading the other one. However, under situations where interactions are bi-directional, the PSI fails in describing the directionality.

The repertoire of the non-linear measures of connectivity involves methods derived from theory of chaotic systems and these based on consideration of probability distribution. Generalized Synchronization (GS) quantifies how well one can predict the trajectory in phase space of the given systems knowing the trajectory of the another; or alternatively, it quantifies how neighborhoods in one attractor maps into the other (Quiroga et al., 2002). Synchronization likelihood (SL) (Stam and van Dijk, 2002), similarly to generalized synchronization, is based on the theory of chaotic systems and makes use of the embedding theorem. SL is defined as the conditional likelihood that the distance between embedded vectors *x* and *y* will be smaller than some cutoff distance. The concept of SL is closely related to the definition of the mutual information (MI).

There are several connectivity measures, which are based on the concept of entropy and involve calculation of probability distributions of given time series e.g., Kulback-Leibler entropy or joint probability distributions e.g., Mutual Information (MI) and Transfer Entropy (TE). TE was introduced by Schreiber (2000) who proposed to use the Kullback-Leibler entropy to quantify a deviation of the transition probabilities from the generalized property of a stationary finite-order Markov processes evolving in time. Non-linear connectivity measures are bivariate except of TE, which was extended to multivariate version by Montalto et al. (2014), so they suffer from common source effect as all bivariate measures.

## Multivariate Measures of Connectivity

### Partial Coherences

EEG is recorded from multiple channels and some of them may be connected directly and some connections can be indirect. To distinguish between these situations partial coherences were introduced. The computation of partial coherence involves calculation of cross-spectral matrix **S**(*f*) between all channels and relies on subtracting from given two channels influences from all other channels under consideration. It is similar to ordinary coherence, but it is nonzero only for direct relations between channels. If a signal in a given channel can be explained by a linear combination of some other signals in the set, the partial coherence between them will be low.

### MVAR Model and Granger Causality

Multivariate Autoregressive Model (MVAR) for *k* channels can be expressed by the formula:

$$X\left(t\right)=\sum_{j=1}^{p}A\left(j\right)X\left(t-j\right)+E\left(t\right).$$ (1)

Where ***X*** (*t*) is a vector of signals, **A**(*j*) is a *k*×*k* matrix of model coefficients, **E**(*t*) is a *k*-size vector of white noises, *p*—model order (the number of samples we take into account in the regression).

Granger causality principle is equivalent to 2-channels MVAR, namely it states that for two time series, if the variance of the prediction error for the second time series is reduced by including past measurements from the first time series in the linear regression model, then the first time series can be said to cause the second time series (Granger, 1969). Connectivity measures Directed transfer Function (DTF) and Partial Directed Coherence (PDC) are based on the Granger causality principle extended to an arbitrary number of channels.

### Multivariate Measures of Effective (Causal) Connectivity

In the frequency domain MVAR can be expressed as a black-box model with noises on input and time series on the output. In the transfer function of the model **H**(*f*) = **A** ^−1^(*f*) the relations between the signals are incorporated.

DTF is formulated in terms of matrix **H**(*f*) elements (Kaminski and Blinowska, 1991):

$${\text{DTF}}_{ij}\left(f\right)=\frac{{\left|H_{ij}\left(f\right)\right|}^{2}}{\sum_{m=1}^{k}{\left|H_{im}\left(f\right)\right|}^{2}}.$$ (2)

and describes causal influence of channel *j* on channel *i* at frequency *f* normalized in respect of inflows to the destination channel *i*.

The modifications of DTF include NDTF (non-normalized DTF), which is directly proportional to the strength of coupling in the given system (Kaminski et al., 2001) and dDTF (direct DTF), which does not show cascade, but only direct flows (Korzeniewska et al., 2003).

Another multivariate estimator based on MVAR is Partial Directed Coherence (PDC) expressing direct interactions (Baccala and Sameshima, 2001):

$${\text{PDC}}_{ij}\left(f\right)=\frac{A_{ij}\left(f\right)}{\sqrt{a_{j}^{*}\left(f\right)a_{j}\left(f\right).}}$$ (3)

where *A*_*ij*_ (*f*) denotes an element of Fourier transformed MVAR coefficients *A*(*t*). The **a**_*j*_ (*f*) denotes the *j*th column of the matrix *A* (*f*), and an asterisk marks the operation of complex conjugation and transposition. Unlike DTF, PDC value shows a ratio between transmission from channel *j* to channel *i* and the summarized outflow from channel *j*, so it tends to emphasize sinks rather than sources. In practice PDC power spectrum weakly depends on frequency and does not have a direct correspondence to the power spectra of the channels of a process.

More comprehensive description of directed connectivity measures may be found in Blinowska (2011). DTF and PDC are available in major toolboxes for biomedical signal analysis. Other connectivity estimators based on Granger causality: Granger Causality Index and (GGC) Granger-Geweke Causality are not widely used for EEG analysis.

### Time-Varying Connectivity

The information processing in brain is a dynamic process of short time scale and EEG accompanying it is a time-varying signal. Its analysis requires use of short data epochs. Fitting of MVAR model to the data epochs is limited by the fact that the number of model parameters has to be preferably one magnitude smaller than the number of the data points. Namely: *pk* ^*2*^ *≤* 0.1*N*_*S*_ for *k* = *pk/N*_*S*_ where *p* is the model order, *k* number of channels, and *N*_*S*_ is the epoch length.

In order to overcome this limitation the short-time DTF (SDTF) was introduced in the following way. We divide a nonstationary recording into shorter time windows, short enough to treat the data within a window as quasi-stationary, but long enough to capture the slowest considered frequency. We calculate the covariance matrix of the model for each short window and then we perform ensamble averaging over realizations of the process. Note, that the averaging concerns correlation matrices for short data windows - data are not averaged in the process. By application of the sliding window we can obtain time-varying MVAR and hence short-time DTF (SDTF) yielding directed dynamic connectivity (Blinowska and Zygierewicz, 2021). The examples of the animations of EEG propagation obtained by means of SDTF for: movement, Constant Attention Test and working memory task may be found at http://fuw.edu.pl/∼kjbli.

The alternative method of getting time-varying connectivity is Kalman filter algorithm. However this method is much more computationally demanding (Kaminski et al., 2010) and is not suitable for a big number of channels.

## Comparison of Connectivity Measures

### Influence of Noise

EEG signals are characterized by a strong noise admixture. The influence of noise on non-linear and linear connectivity estimators was studied by Netoff et al. (2006). They reported low robustness to noise of non-linear measures in comparison to the linear ones. The conclusion of the seminal study by Pereda et al. (2005) was that linear measures of connectivity are superior in comparison to non-linear ones in respect of robustness to noise and authors recommended the use of non-linear methods only in case when the non-linearity was found in the data or relations between them. The test for non-linearity relies on comparison of the results obtained by original data with surrogate data (signals with randomized phases). For resting state EEG the degree of non-linearity was estimated by means of surrogate data as 4.4% (Päeske et al., 2018). This finding was close to the one of Orgo et al. (2017), who reported 6.1% and to the result of (Breakspear and Terry, 2002) who detected statistically significant evidence of non-linear interactions as 4.8%. The presence of non-linearity were found in certain epochs of epileptic seizure (Pijn et al., 1997). It is worth mentioning that even for nonlinear time series the directed connectivity is correctly estimated by Granger-based multivariate estimators (Winterhalter et al., 2005). Connectivity measures based on MVAR are robust to noise, since they explicitly assume its existence. It was demonstrated by simulation that even in case of noise three times as big as EEG signal the connectivity scheme was well reproduced by DTF (Kaminski and Blinowska, 1991).

Considering information processing in brain interesting nonlinear measures are the ones estimating cross-frequency coupling and phase-amplitude coupling (Blinowska and Zygierewicz, 2021). However, calculation of these measures requires high quality data.

### Volume Conduction

Volume conduction concerns effects of propagation of the electromagnetic field on the results of EEG analysis (Hassan and Wendling, 2018; Marinazzo et al. (2019); Van de Steen et al., 2019). Several methods were developed to project the activity recorded from scalp to the source space, e.g.,: see review by: Grech et al. (2008); among them Loreta (Frei et al., 2001) gained popularity. However solution of the inverse problem is not unique, it involves certain assumptions and it might disturb the phases between the signals. Electromagnetic field propagates with a speed of light, so volume conduction does not produce phase differences between EEG channels. Therefore its influence is to the large degree mitigated in case of the connectivity measures based on the phase difference—among them DTF and PDC (Kaminski and Blinowska, 2014). Still, there might be some effects connected with mixing of sources activity, but they are limited by the fact that the electric field of the dipole layer decays practically to zero at the distance of ∼7 cm (Nunez and Srinivassan, 2006). Connectivity results accompanying finger movement based on the “source” data obtained by application of Loreta were compared with those based on scalp signals (Kaminski and Blinowska, 2018). It was found that in case of cortex projected signals the changes in connectivity in time and space during the movement were hardly observed, which might be due to disturbance of phases in computation of inverse solutions. In case of scalp electrodes the connectivity estimated by means of DTF exactly reflected known topographic effects of desynchronization and resynchronization in relevant brain structures. The excellent topographic agreement of connectivity results obtained by DTF and based directly on scalp recordings, with anatomical, physiological and imaging evidence (Kaminski and Blinowska, 2018) indicate that for estimators based on phase differences the projection to the source space seems not to be necessary, however debate concerning the mitigation of volume conduction effects is still going on.

### Effect of Common Source

**FIGURE 1:**
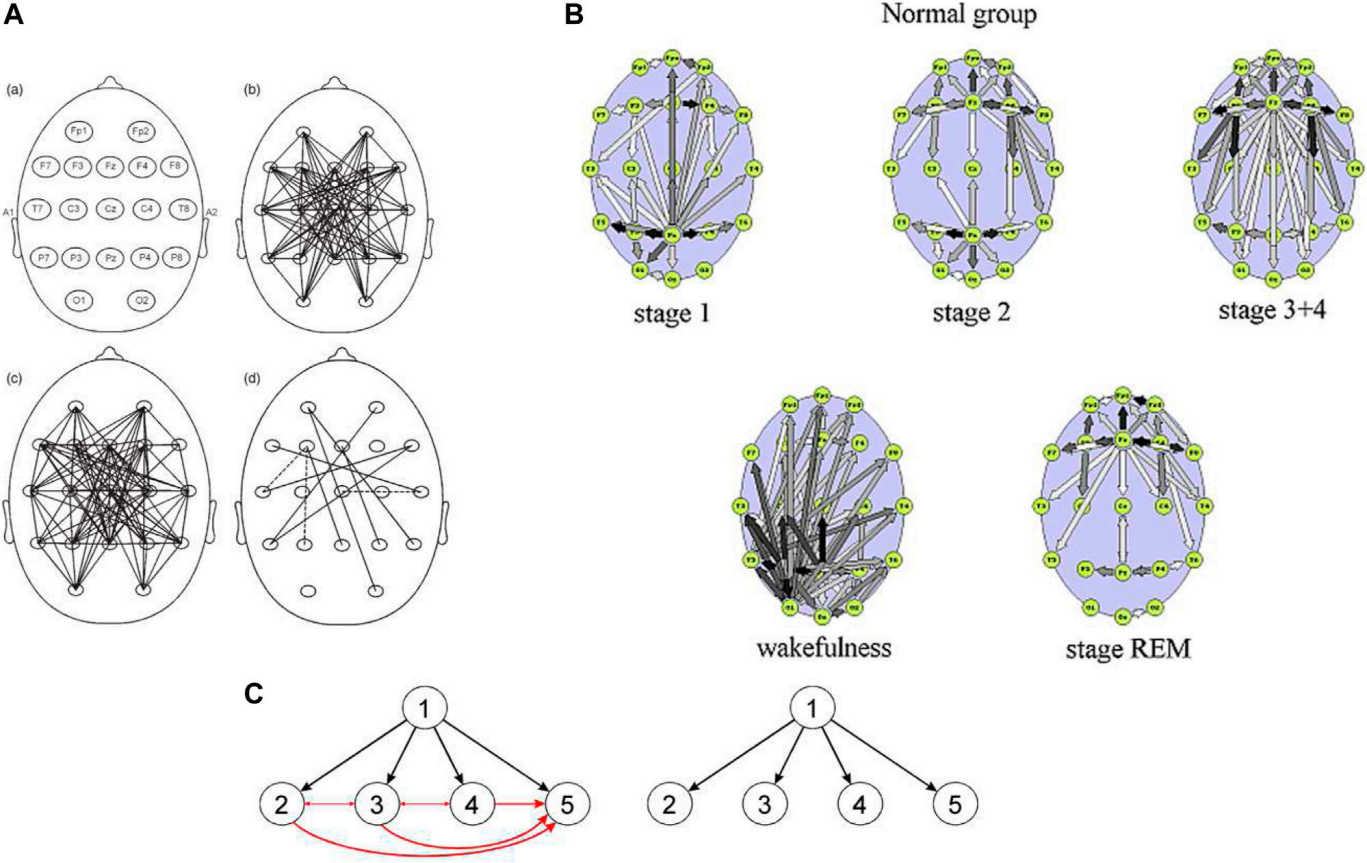
Connectivity patterns during sleep. **(A)** Connectivity obtained by means of bivariate measure (SL) in delta band (slow wave sleep- SWS); b) for healthy subjects, c) for depressed subjects, d) differences between healthy and depressed subjects. (Leistedt et al., 2009). **(B)**. Connectivity patterns obtained in three sleep stages averaged over 9 healthy subjects (Kaminski et al., 1997). Stage 3 (SWS) corresponds to image **A** picture c), Adapted from (Kaminski et al., 1997; Leistedt et al., 2009) with permission. **(C)—**image at left shows simulated scheme of propagation (this kind of scheme is obtained by multivariate methods: DTF or PDC). Image at the right represents the connectivity scheme obtained by bivariate methods.

The effect which may critically corrupt connectivity measures is the common drive effect. Namely, if the EEG activity from a given source is detected at, say *N* electrodes, bivariate measure will show not only *N* true connections outgoing from the source but also *N*(*N*−1)/2 connections between all electrodes recording the source activity (see insert in Figure 1). In this way the false connections outnumber the true ones, since they increase as *N* ^2^ (Blinowska et al., 2004) (Kaminski & Blinowska, 2013). Because of the common drive effect connectivity obtained by means of all bivariate measures yield dense, disorganized patterns, contrary to multivariate measures such as DTF and PDC which show sparse EEG networks.

As an example may serve the determination of the connectivity during sleep. In Figure 1 EEG connectivity patterns obtained during sleep are shown: **A-** by means of bivariate measure—SL (Leistedt et al., 2009), and **B—**by multivariate measure—DTF (Kaminski et al., 1997). Networks yielded by means of SL look almost random, the authors qualified them as “small world”. It is difficult to unravel obtained patterns and understand why such disorganized pattern occurs in case of slow wave sleep (Figure 1A) when whole brain works synchronously. The networks shown in **B** are characterized by distinct features compatible with the physiological knowledge. In stage 1 pattern is similar to the one connected with wakefulness, but more ordered, in stage 2 two main sources of activity are connected with two sources of spindles (frontal and posterior), in stage 3, characterized by a global synchronization in delta band, there is practically one source located above corpus callosum, where from all major neural tract diverge. It is easy to see that multiple connections in Figure 1A are due to common source effect, which produces multiple false connections. More comprehensive comparison of networks obtained by means of bivariate and multivariate connectivity measures may be found in (Blinowska and Kaminski, 2013).

## Post-Processing of Connectivity Results

In order to quantify the results obtained by means of bivariate measures usually graph analysis (GA) is applied in its classical binary form. It assumes a network consisting of nodes (corresponding to the electrodes) and edges (connections). Networks should be fully connected (every node with every other node). Connections above some threshold are set to equal values. Mostly used parameters describing a network are clustering coefficient, path length, node degree, global efficiency. Clustering coefficient (CC) is a measure of local interconnectedness of a graph, path length (PL) is the shortest path between two nodes expressed as the number of traversed nodes. So-called “small-world” (SW) networks are characterized by a high CC and a short PL. Common practice is that to distinguish between the experimental conditions or groups of subjects CC and PL are determined and “small world” topology is searched. However the results obtained by different researchers are often divergent as is the case for schizophrenia (Rutter et al., 2013) and Alzheimer disease (Tijms et al., 2013). The information obtained by GA binary networks is strongly dependent on the density of nodes and setting of the thresholds. It has very global character neglecting weights and topographical specificity of the networks. The results of binary GA hardly conform with known anatomical and physiological evidence (Kaminski and Blinowska, 2018).

In the last years we can observe accumulating criticism concerning binary GA application to EEG/MEG signals: e.g.,: (Hilgetag and Goulas, 2016), (Papo et al., 2016). The evidence that binary conventional GA are extreme simplification of brain networks was brought by the tract-tracing experiments indicating that large-scale neuronal networks of the brain are arranged as globally sparse hierarchical modular networks e.g., (Oh et al., 2014). The experts in “smallworldness” had to admit that binary unweighted networks are not adequate for description of brain networks (Basset and Bullmore, 2017).

In fact, multivariate methods such as DTF since nineties of 20th century provided in multiple applications sparse, weighted, directed networks compatible with modern evidence of topologically segregated and anatomically localized networks (Blinowska and Zygierewicz, 2012; Blinowska and Zygierewicz, 2021). This kind of connectivity patterns may be quantified by means of more advanced version of GA based on assortative mixing (Newman and Girvan, 2004). This approach takes into account weights and directions of connections. The connectivity matrix *E*_*kl*_, is defined to be a fraction of edges in a network that connects a node of group *k* to one of group *l*. Indexes *k* and *l* do not necessary refer to the channels, but to the modules (region of interest—ROIs) defined in the framework of assortative mixing. For directed networks matrix *E*_*kl*_ is asymmetric. Mixing is highly assortative when the diagonal elements of matrix *E*_*kl*_ are significantly higher than the off-diagonal ones. It corresponds to the situation of strongly connected modules, with weaker bonds between them.

**FIGURE 2:**
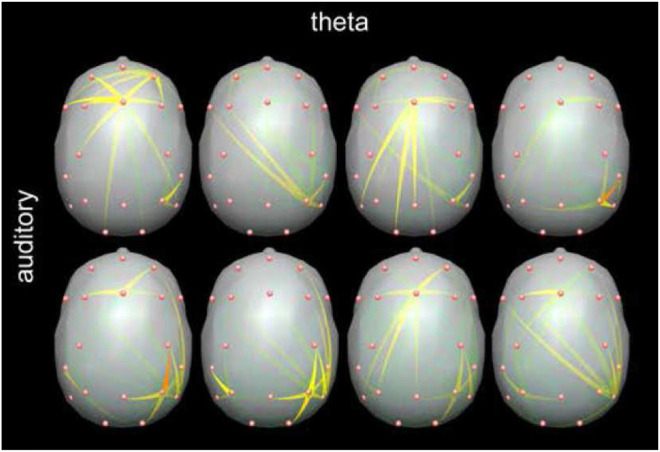
Snapshots from animation showing propagation in the teta frequency band during working memory task obtained by time-varying DTF (SDTF). The time lapse between the pictures is 0.75 s. The color scale shows flow intensity (red is the strongest). The engagement of the frontal and posterior/temporal regions can be observed. Long-range transmissions from frontal to posterior locations and vice-versa occur intermittently. Adapted from Kaminski et al., 2018.

Assortative mixing approach was applied for quantification of networks active during visual Working Memory task (Blinowska et al., 2013) and comparison of networks patterns active during visual and auditory Working Memory tasks (Kaminski et al., 2018). During both kinds of task similar patterns of connectivity were found with tightly connected modules in frontal and parietal (visual task) and parietal/temporal locations. These modules were connected reciprocally by weaker links acting intermittently (Figure 2). The location of active modules was coincident with fMRI imaging (Brzezicka et al., 2011).

## Conclusion

The accumulated body of evidence points out that bivariate connectivity measures do not represent adequately the connectivity structure of brain networks, since the obtained network patterns are blurred by the large number of spurious connections. The notion of “smallworldness” is too general and imprecise to describe in satisfactory way the topologically segregated and physiologically specific brain networks. The problem standing now before a scientific milieu concerns how to reconcile most popular today approach of connectivity assessment with physiological evidence showing modular structure of networks, weights heterogeneity and directionality of information transfer. Our aim should be to find richer and more meaningful model describing brain connectivity. The questions and challenges to be faced include: 1) which connectivity measure is the most appropriate to solve a given problem? 2) which approach may overcome binary graph limitations towards incorporating weights and directionality to describe topologically specific connectivity structure? The tools to overcome the above mentioned limitations are at hand; they involve application of multivariate measures of connectivity and more advanced graph analysis methods (Kaminski and Blinowska, 2017; Imperatori et al., 2019).

## Data Availability

The original contributions presented in the study are included in the article/Supplementary Material, further inquiries can be directed to the corresponding author.
